# Kalman Filter-Based Epidemiological Model for Post-COVID-19 Era Surveillance and Prediction

**DOI:** 10.3390/s25082507

**Published:** 2025-04-16

**Authors:** Yuanyou Shi, Xinhang Zhu, Xinhe Zhu, Baiqi Cheng, Yongmin Zhong

**Affiliations:** School of Engineering, RMIT University, Melbourne, VIC 3000, Australia; yuanyou.shi@rmit.edu.au (Y.S.); s3933085@student.rmit.edu.au (X.Z.); xinhezhu@ncut.edu.cn (X.Z.); sigurd.cbq425@gmail.com (B.C.)

**Keywords:** COVID-19, antibody-acquired, viral variants, SEIRD model, extended Kalman filter

## Abstract

In the post-COVID-19 era, the dynamic spread of COVID-19 poses new challenges to epidemiological modelling, particularly due to the absence of large-scale screening and the growing complexity introduced by immune failure and reinfections. This paper proposes an AEIHD (antibody-acquired, exposed, infected, hospitalised, and deceased) model to analyse and predict COVID-19 transmission dynamics in the post-COVID-19 era. This model removes the susceptible compartment and combines the recovered and vaccinated compartments into an “antibody-acquired” compartment. It also introduces a new hospitalised compartment to monitor severe cases. The model incorporates an antibody-acquired infection rate to account for immune failure. The Extended Kalman Filter based on the AEIHD model is proposed for real-time state and parameter estimation, overcoming the limitations of fixed-parameter approaches and enhancing adaptability to nonlinear dynamics. Simulation studies based on reported data from Australia validate the AEIHD model, demonstrating its capability to accurately capture COVID-19 transmission dynamics with limited statistical information. The proposed approach addresses the key limitations of traditional SIR and SEIR models by integrating hospitalisation data and time-varying parameters, offering a robust framework for monitoring and predicting epidemic behaviours in the post-COVID-19 era. It also provides a valuable tool for public health decision-making and resource allocation to handle rapidly evolving epidemiology.

## 1. Introduction

As of the end of 2024, with more than 99% of the global population having been vaccinated or recovered from infection, the social health impact caused by the COVID-19 pandemic has gradually levelled off, according to an online database [1]. Amidst this global upheaval, COVID-19-related deaths remain significantly higher than those caused by influenza [2], placing a substantial burden on healthcare resources. Moreover, the potential crisis persists, as several emerging variants exhibit immune escape characteristics, particularly in cases of prolonged infection [3,4]. Compounding these challenges, the availability of infection monitoring data has become increasingly scarce, limiting the ability to accurately assess and respond to the evolving pandemic [5]. These factors underscore the urgent need for adaptable and efficient modelling approaches to the prediction and management of this dynamic landscape.

During the COVID-19 pandemic, various modelling methods were proposed to investigate the propagation of COVID-19, which were generally classified into two categories: agent-based modelling and compartmental modelling. Agent-based models capture the dynamic propagation behaviours of COVID-19 by simulating interactions among micro-level agents, but they face challenges due to computational complexity and prediction robustness [6]. In contrast, compartmental models categorise the community into several compartments using differential equations for efficient computation [7]. The SIR (susceptible, infected, and recovered) model represents susceptible, infected, and recovered populations [8]. The SEIR (susceptible, exposed, infected, and recovered) model improves the SIR model by introducing the exposed compartment to account for the disease’s incubation period [9]. Since the ferocity of the epidemic has claimed many lives, its lethality cannot be ignored. Accordingly, the SEIRD (susceptible, exposed, infected, recovered, and deceased) model introduces the deceased compartment into the SEIR model to account for the deceased population [10]. The above models rely on comprehensive report data and well-defined parameters to accurately describe disease dynamics. However, due to the discontinuation of large-scale screening programmes, the report data on COVID-19 are limited to fewer compartments, leading to degraded modelling accuracy [11]. Moreover, these models cannot reflect the effects of vaccinations and viral variants in the post-COVID-19 era.

To address the problem caused by limited report data for COVID-19 prediction and analysis, researchers have turned to alternative data sources. Badr et al. leveraged mobility data to infer population movement patterns, enabling the estimation of disease transmission rates in the absence of comprehensive case tracking [12]. However, since this approach primarily mitigates data shortages through alternative datasets, it fails to provide reliable data for model calibration. Hospital admissions offer a stable data source to support accurate posterior correction for COVID-19 estimation.

On the other hand, continuous mutations in SARS-CoV-2 have also complicated COVID-19 modelling, which is required to characterise the immune effect [13]. Given the critical role of immunity in determining disease propagation and control, understanding the interaction between infection-derived and vaccination-derived immunity is important. The human body initiates the production of Immunoglobulin M (IgM) as an early response to the virus, succeeded by Immunoglobulin G (IgG), which provides prolonged immune defence, preventing potential reinfection by the same virus strain [14]. This indicates that both vaccination and infection result in a comparable concentration and duration of IgG-mediated immune protection, regardless of how it is acquired [15]. However, research shows that individuals who recovered from infection and developed IgG antibodies may still exhibit IgM antibodies in their serum tests after a certain period [5], unveiling that such individuals may not possess the anticipated permanent resistance to virus variants [16]. This indicates that immunity acquired through infection or vaccination does not guarantee permanent resistance to emerging viral variants. The uncertainty of immune durability and the risks from variant infections highlight the need to integrate immune failure mechanisms into COVID-19 propagation models. Resolving these complexities is crucial for accurately capturing disease dynamics in the post-COVID-19 era and enhancing prediction reliability.

To study the effect of immunity, the SEIRS (susceptible, exposed, infected, and temporarily recovered) model incorporates an immune failure rate into the SEIR model to account for reinfections due to the evasion of natural immunity in recovered patients who are not vaccinated [17]. However, this model does not account for the high risk of mortality associated with COVID-19 and immune failure due to the vaccine’s ineffectiveness and fading memory, as well as viral variants. Zhu et al. improved the SEIRS model by introducing the deceased compartment [18]. However, similar to the SEIRS model, this approach still cannot account for reinfections caused by immune failure due to the vaccine’s ineffectiveness and fading memory, as well as viral variants. In the post-COVID-19 era, a high percentage of the population has been vaccinated, and both recovered and vaccinated individuals possess antibodies for immunity, thus reducing the reinfection risk. Zhu et al. studied the reinfection effect in the post-COVID-19 era [19]. This approach considers the immune effects of recovered and vaccinated individuals separately. However, in reality, it is difficult to distinguish between the immunity of recovered and vaccinated individuals, as both possess antibodies, leading to combined immunity. Therefore, it is necessary to place recovered and vaccinated individuals into one antibody group to study their combined immune effect in the post-COVID-19 era with vaccinations and viral variants.

Despite the growing recognition of immune failure mechanisms and their impact on COVID-19 dynamics, most modelling studies predominantly rely on approaches with fixed-parameter modelling. This reliance limits their ability to accommodate time-dependent variations in a dynamic transmission process, particularly the time-varying infection rate, as described above. Furthermore, these studies are also mainly conducted in an offline manner [11,13,20,21,22,23]. Online estimation schemes, such as Recursive Least Squares (RLS), Kalman Filtering (KF), and Extended Kalman Filtering (EKF) [24,25,26], are indispensable to modelling the dynamic behaviours of COVID-19 transmission and its parameter variations.

The RLS method is commonly used to estimate the state of an infectious disease [21]. Similarly, KF refines RLS using state-space equations, allowing real-time updates of disease spread predictions under noisy or incomplete data. Recent studies have utilised KF to estimate the growth rate of infected individuals across 124 countries [25]. Additionally, KF has also been employed to predict the trajectory of infection cases over a 30-day period in India [23]. However, both RLS and KF are constrained to linear systems, whereas most epidemiological models for COVID-19 prediction are inherently nonlinear. EKF addresses this limitation by expanding KF to nonlinear systems via the linearisation of first-order Taylor series expansion. EKF has been used to estimate compartmental model dynamics and their associated model parameters for COVID-19 prediction [19,21,26]. Nevertheless, previous studies largely rely on conventional SIR or SEIR models and assume the availability of comprehensive report data on COVID-19. However, with large-scale screening no longer accessible in the post-COVID-19 era, the limited availability of infection and vaccination statistics may yield biases in estimation results.

In this paper, we propose an AEIHD (antibody-acquired, exposed, infected, hospitalised, and deceased) model to address the limitations of traditional epidemiological models in the post-COVID-19 era. This model removes the susceptible compartment and merges the recovered and vaccinated compartments into a single “antibody-acquired” compartment. A hospitalisation compartment is also introduced to monitor severe cases, offering critical data for estimating the total number of infections, particularly in the absence of large-scale screening programmes. To account for reinfections caused by immune failure, an antibody-acquired infection rate is incorporated, enabling the evaluation of its impact on epidemic progression. Furthermore, the model employs EKF to estimate real-time states and parameters, overcoming the constraints of fixed-parameter approaches and enhancing its adaptability to nonlinear dynamics. Using reported data from Australia, the AEIHD model was validated through simulation studies, demonstrating its capability to provide accurate and timely estimations of COVID-19 transmission dynamics.

Compared to the existing models, especially the SEIRS model, the novelties of the proposed AEIHD model include the following: (i) Since the ferocity of COVID-19 has claimed many lives, its lethality cannot be ignored. The proposed AEIHD model introduces the death compartment to account for the lethality of COVID-19, while the SEIRS does not consider the lethality effect. (ii) In the post-COVID-19 era, both recovered and vaccinated individuals possess antibodies for immunity. Given the difficulty in distinguishing between the immunity of recovered and vaccinated individuals, the proposed AEIHD model combines recovered and vaccinated individuals into one common antibody group to study their combined immune effect. It further introduces the antibody-acquired infection rate to account for reinfections caused by immune failure due to the vaccine’s ineffectiveness, immunity evasion, and viral variants. However, the SEIRS model only considers reinfections caused by natural immunity’s evasion in recovered patients, without considering the effects of vaccination and viral variants in the post-COVID-19 era. (iii) In the post-COVID-19 era, as most people have acquired antibodies through vaccination or natural infection, the susceptible population is very small, and thus, its effect can be ignored. Accordingly, the AEIHD model removes the susceptible compartment, while the SEIRS model considers the susceptible compartment as an important factor, failing to reflect this population change in the post-COVID-19 era. (iv) In the post-COVID-19 era, the reported data on COVID-19 are limited to fewer compartments, degrading the prediction accuracy of the SEIRS model. Since hospital admissions are a reliable data source, the proposed AHEID model introduces the hospitalised compartment to account for severe cases reflected by hospital admissions to achieve modelling reliability. In general, the proposed AHEID model can account for transmission characteristics in the post-COVID-19 era, while the SEIRS cannot.

## 2. Methodology

### 2.1. Epidemiological Model

**Figure 1:**
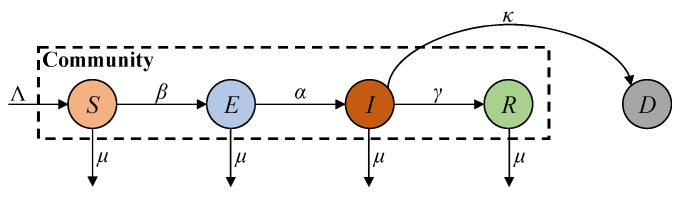
SEIRD model transmission framework.

The SEIRD compartmental model is a widely used framework for studying the dynamics of infectious diseases (such as COVID-19) during their early stages. The majority of the population in the community initially consists of susceptible individuals who are at risk of infection through exposure to the virus [27,28]. Here, the population is categorised into five distinct compartments: susceptible, exposed, infected, recovered, and deceased, and their transmission dynamics are governed by the framework shown in Figure 1.

In Figure 1, *S*, *E*, *I*, *R*, and *D* represent the susceptible, exposed, infected, recovered, and deceased compartments, respectively; Λ denotes the number of natural births; *α* and *β* denote the infection growth rate (i.e., the inverse of the incubation period) and infection rate; *γ* denotes the recovery rate from infection to recovery (i.e., the product of the inverses of the recovery time and the population ratio); and *μ* and *κ* denote the death rates related to natural causes and infection, respectively. Additionally, the deceased compartment is a terminal state that does not influence the virus transmission process apart from natural deaths and is therefore considered outside of community dynamics. Based on this framework, the SEIRD model can be formulated as the following set of differential equations:

(1) $$\left\{\begin{array}{l} \frac{dS(t)}{dt}=\Lambda -\beta I(t)S(t)-\mu S(t)\\ \frac{dE(t)}{dt}=\beta I(t)S(t)-\alpha E(t)-\mu E(t)\\ \frac{dI(t)}{dt}=\alpha E(t)-(\gamma +\kappa )I(t)-\mu I(t)\\ \frac{dR(t)}{dt}=\gamma I(t)-\mu R(t)\\ \frac{dD(t)}{dt}=\kappa I(t) \end{array}\right.$$

In this paper, we propose a new AHEID model to reflect these characteristics in the post-COVID-19 era. This model improves the SEIRD model by removing the susceptible compartment and combining the vaccinated and recovered individuals into a single antibody-acquired compartment, *A*, to study their combined immune effect in the post-COVID-19 era with vaccinations and viral variants. The hospitalised compartment, *H*, is incorporated into the AHEID model to track severe symptomatic cases reported by hospital admissions. Additionally, the distinct recovery rates for infected and hospitalised patients are also introduced to model their transitions to the *A* compartment. The AEIHD model can be formulated as a combination of differential equations:

(2) $$\left\{\begin{array}{l} \frac{dA(t)}{dt}=\Lambda -\beta_{A}I(t)A(t)+\gamma_{I}I(t)+\gamma_{H}H(t)-\mu A(t)\\ \frac{dE(t)}{dt}=\beta_{A}I(t)A(t)-\alpha E(t)-\mu E(t)\\ \frac{dI(t)}{dt}=\alpha E(t)-(\gamma_{I}+\eta )I(t)-\mu I(t)\\ \begin{array}{l} \frac{dH(t)}{dt}=\eta I(t)-(\gamma_{H}+\kappa_{H})H(t)-\mu H(t)\\ \frac{dD(t)}{dt}=\kappa_{H}H(t) \end{array} \end{array}\right.$$

where *A*, *E*, *I*, *H*, and *D* denote the antibody-acquired, exposed, infected, hospitalised, and deceased compartments; *β_A_* denotes the antibody-acquired infection rate of antibody-acquired individuals; *η* denotes the severe symptomatic rate; *γ_I_* and *γ_H_* represent the recovery rates from the infected and hospitalised compartments, respectively; and *κ_H_* represents the deceased rate from the hospitalised compartment. Notably, introducing a natural birth rate into the *A* compartment inevitably results in the inclusion of individuals who have neither been vaccinated nor acquired immunity through infection. However, the number of such individuals is relatively small, and introducing a separate susceptible compartment for them would significantly increase the model’s complexity without substantially affecting the overall epidemic dynamics. Moreover, considering both natural births and deaths is essential for maintaining the mathematical stability of the population structure. Assigning newborns directly to the *A* compartment serves as a reasonable approximation in the context of the post-COVID-19 era. The active total population in the community always satisfies the following condition:

(3) N(t)=A(t)+E(t)+I(t)+H(t)

where *N*(*t*) denotes the active population within the community who can participate in the transmission process. This population does not include deceased individuals.

**Figure 2:**
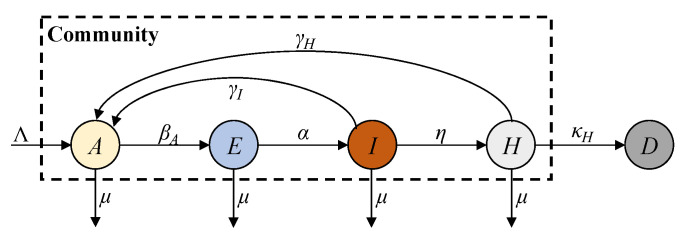
AEIHD model transmission framework.

The AEIHD model framework is shown in Figure 2. The individuals with acquired antibodies may move to the exposed compartment at the antibody-acquired infection rate *β_A_* after contact with an infectious source. Exposed individuals develop symptoms and are transferred to the infected compartment at rate *α*. The infected compartment has two possible outcomes: either infected people recover and transition to the antibody-acquired compartment at rate *γ_I_*, or they develop severe symptoms and are hospitalised at rate *η*. Similarly, hospitalised patients have two possible outcomes: either they recover and transition to the antibody-acquired compartment at rate *γ_H_*, or they are transferred to the deceased compartment with the death rate *κ_H_*.

### 2.2. Stability Analysis

In this section, the stability of the AEIHD model is analysed in terms of non-negativity, boundedness, and disease-free and endemic equilibria. These theoretical validations affirm the model’s consistency with real-world epidemic dynamics and its applicability in the post-COVID-19 era.

#### 2.2.1. Non-Negativity and Boundedness

**Lemma** **1.** *Given the non-negative initial conditions A(0) ≥ 0, E(0) ≥ 0, I(0) ≥ 0, H(0) ≥ 0, and D(0) ≥ 0, N(t) remains non-negative for all t ≥ 0.*

**Proof.**  The positive terms are removed from the first equation of Equation (2) to construct the following inequality:

(4) $$\frac{dA(t)}{dt}\geq -g(t)A(t)$$

where *g*(*t*) = *β_A_I*(*t*) + *µ*.

Integrating both sides of (4) yields

(5) $$A(t)\geq A(0)e^{-\int_{0}^{t}g(s)ds}\geq 0$$

Thus, *A*(*t*) remains non-negative for all *t* ≥ 0. Similarly, the non-negativity of all other compartments can also be proven. Consequently, the variation in the total population is also non-negative, i.e.,

(6) N(t)≥0

This completes the proof of Lemma 1. □

**Lemma** **2.** *Given the above non-negative initial conditions, N(t) is bounded and N(t) ≤* Λ*/μ for all t ≥ 0.*

**Proof.**  From (3), it follows that

(7) $$\frac{dN(t)}{dt}=\frac{dA(t)}{dt}+\frac{dE(t)}{dt}+\frac{dI(t)}{dt}+\frac{dH(t)}{dt}$$

By summing all equations in (2) together, we have

(8) $$\frac{dN(t)}{dt}=\Lambda -\mu N(t)-\kappa_{H}H(t)$$

Then, *–κ_H_H*(*t*) is removed to construct the following inequality:

(9) $$\frac{dN\left(t\right)}{dt}+\mu N\leq \Lambda$$

We rewrite (9) as

(10) $$\frac{d}{dt}\left\{N\left(t\right)e^{\mu t}\right\}\leq \Lambda e^{\mu t}$$

Integrating both sides of (10) generates

(11) $${\left.e^{\mu t}N\left(t\right)\right|}_{0}^{t}\leq \int_{0}^{t}e^{\mu t}\Lambda dt$$

From (11), we readily have

(12) $$N\left(t\right)\leq \frac{\Lambda }{\mu }+\left(N(0)-\frac{\Lambda }{\mu }\right)e^{-\mu t}$$

Thus, we have

(13) $$N\left(t\right)\leq \max\left(N(0),\frac{\Lambda }{\mu }\right)$$

From (13), it is evident that *N*(0) ≤ Λ/*μ* is a sufficient condition to achieve

(14) $$N\left(t\right)\leq \frac{\Lambda }{\mu }$$

Finally, combining (6) and (14), we derive

(15) $$0\leq N\left(t\right)\leq \frac{\Lambda }{\mu }$$

This completes the proof of Lemma 2. □

#### 2.2.2. Basic Reproduction Number

Let $x={\left[A,E,I,H,D\right]}^{T}$ denote the state vector of the system based on the AEIHD model. The initial state vector ***x***^0^ represents a population primarily comprising the vaccinated or recovered individuals in compartment *A*^0^, i.e.,

(16) $$x^{0}=\left(A^{0},0,0,0,0\right)$$

where $A^{0}=\Lambda /\mu$ denotes the antibody-acquired compartment at the disease-free equilibrium, which serves as the starting point for the stability analysis.

The derivatives of the exposed and infected compartments can be rewritten in a matrix form as

(17) $$G^{\prime }=U\left(G\right)-V\left(G\right)$$

where $G={\left[E,I\right]}^{T}$, $U(G)={\left[\beta_{A}IA^{0},0\right]}^{T}$, and $V(G)={\left[(\alpha +\mu )E,-\alpha E+(\gamma I+\eta +\mu )I\right]}^{T}$.

The partial derivatives of U and V at the initial state can be obtained:

(18) $$U=\left(\begin{array}{cc} 0 & \beta A^{0}\\ 0 & 0 \end{array}\right),\;\mathrm{and}\;V=\left(\begin{array}{cc} \alpha +\mu & 0\\ \alpha & \gamma_{I}+\eta +\mu \end{array}\right)$$

Using the next-generation matrix method [28], the basic reproduction number $R_{0}$ is given by the spectral radius (i.e., the largest eigenvalue modulus) of the next-generation matrix ***UV***^−1^, i.e.,

(19) $$R_{0}=\frac{\Lambda \alpha \beta_{A}}{\mu \left(\alpha +\mu \right)\left(\mu +\eta +\gamma_{I}\right)}$$

The epidemic is expected to spread if $R_{0}>1$ or tend to end if $R_{0}<1$.

#### 2.2.3. Disease-Free and Endemic Equilibria

In order to provide critical insights into the stability and prolonged dynamics of the epidemic, we analyse the disease-free and endemic equilibria of the proposed AEIHD model. The Jacobian matrix of the AEIHD model is given by

(20) $$J(x)=\left(\begin{array}{ccccc} -\beta_{A}I-\mu & 0 & -\beta_{A}A+\gamma_{1} & \gamma_{H} & 0\\ \beta_{A}I & -\alpha -\mu & \beta_{A}A & 0 & 0\\ 0 & \alpha & -(\gamma_{I}+\eta +\mu ) & 0 & 0\\ 0 & 0 & \eta & -\left(\gamma_{H}+\kappa_{H}+\mu \right) & 0\\ 0 & 0 & 0 & \kappa_{H} & 0 \end{array}\right)$$

**Theorem** **1.** *The AEIHD system at **x***^0^ *will be partially asymptotically stable when $R_{0}<1$. Otherwise, the disease-free equilibrium will not exist.*

**Proof.**  By substituting ***x***^0^ into (20), we obtain

(21) $$J\left(x^{0}\right)=\left(\begin{array}{ccccc} -\mu & 0 & \gamma_{I}-\beta_{A}A^{0} & \gamma_{H} & 0\\ 0 & -\varepsilon_{1} & \beta_{A}A^{0} & 0 & 0\\ 0 & \alpha & -\varepsilon_{2} & 0 & 0\\ 0 & 0 & \eta & -\varepsilon_{3} & 0\\ 0 & 0 & 0 & \kappa_{H} & 0 \end{array}\right)$$

where *ε*_1_ = *α* + *μ*, *ε*_2_ = *μ + γ_I_* + *η*, and *ε*_3_ = *μ* + *γ_H_* + *κ_H_*, and the eigenvalues of ***J*** are computed by

(22) $$\left\{\begin{array}{l} \lambda_{1}=-\mu \\ \lambda_{2}=-\varepsilon_{3}\\ \lambda_{3}=-\frac{1}{2}\left(\varepsilon_{1}+\varepsilon_{2}+\sqrt{\varepsilon_{4}}\right)\\ \begin{array}{l} \lambda_{4}=-\frac{1}{2}\left(\varepsilon_{1}+\varepsilon_{2}-\sqrt{\varepsilon_{4}}\right)\\ \lambda_{5}=0 \end{array} \end{array}\right.$$

where $\varepsilon_{4}={\left(\varepsilon_{1}-\varepsilon_{2}\right)}^{2}+4\varepsilon_{1}\varepsilon_{2}R_{0}$.

When $R_{0}<1$, the eigenvalues *λ*_1:4_ are consistently negative and *λ*_5_ remains zero, which implies that the AEIHD system at the disease-free equilibrium is locally asymptotically stable. In other words, the population state converges to the disease-free equilibrium over time. Conversely, if $R_{0}>1$, *λ*_4_ < 0 cannot be ensured, indicating that disease-free equilibria will not be achieved, raising the potential risk of a pandemic.

The proof of Theorem 1 is completed. □

**Theorem** **2.** *The endemic equilibrium is uniquely defined when* $R_{0}>1$.

**Proof.**  The endemic equilibrium x^*^ = (A^*^, E^*^, I^*^, H^*^, D^*^) satisfies the condition that all derivatives in (2) are zero, i.e.,

(23) $$\begin{array}{l} 0=\Lambda -\beta_{A}A^{*}I^{*}+\gamma_{I}I^{*}+\gamma_{H}H^{*}-\mu A^{*}\\ 0=\beta_{A}A^{*}I^{*}-\alpha E^{*}-\mu E^{*}\\ 0=\alpha E^{*}-(\gamma_{I}+\eta )I^{*}-\mu I^{*}\\ \begin{array}{l} 0=\eta I^{*}-(\gamma_{H}+\kappa_{H})H^{*}-\mu H^{*}\\ 0=\kappa_{H}H^{*} \end{array} \end{array}$$

Solving (23) in terms of $I^{*}$ yields

(24) $$\left\{\begin{array}{l} A^{*}=\frac{\varepsilon_{3}\Lambda +\left(\varepsilon_{3}\gamma_{I}+\gamma_{H}\eta \right)I^{*}}{\varepsilon_{3}\left(\beta_{A}I^{*}+\mu \right)}\\ E^{*}=\frac{\left[\varepsilon_{3}\beta_{A}\Lambda I^{*}+\beta_{A}\left(\varepsilon_{3}\gamma_{I}+\gamma_{H}\eta \right)I^{*2}\right]}{\varepsilon_{1}\varepsilon_{3}\left(\beta_{A}I^{*}+\mu \right)}\\ H^{*}=\frac{\eta I^{*}}{\varepsilon_{3}} \end{array}\right.$$

Substituting (24) into the third equation of (23), we obtain the following quadratic form in terms of I^*^

(25) $$\xi_{2}I^{*2}+\xi_{1}I^{*}=0$$

where

(26) $$\begin{array}{l} \xi_{1}=\mu \kappa_{H}\varepsilon_{1}\varepsilon_{2}\left(1-R_{0}\right)\\ \xi_{2}=\beta_{A}\left[\alpha \left(\varepsilon_{3}\gamma_{I}+\gamma_{I}\eta \right)+\varepsilon_{1}\varepsilon_{2}\varepsilon_{3}\right] \end{array}$$

It is evident that *ξ*_2_ remains positive and *ξ*_1_ remains negative when $R_{0}>1$, so a unique positive solution for I^*^ exists. This indicates a unique endemic equilibrium for $R_{0}>1$. Once the system reaches the endemic equilibrium, the pandemic tends to be stabilised, neither vanishing nor growing exponentially. The above analysis demonstrates that the AEIHD model consistently achieves equilibrium, indicating its inherent convergence.

The proof of Theorem 2 is completed. □

### 2.3. Estimation Method

#### System State and Observation Equation

Using the AEIHD model, a state-space equation is facilitated by applying Euler’s integration, expressed as

(27) $$x_{t+1}=\phi (x_{t})$$

where ϕ(·) denotes a nonlinear function derived from (2), i.e.,

(28) $$\phi (x_{t})=\left\{\begin{array}{l} A_{t}+\Lambda -\beta_{A}A_{t}I_{t}+\gamma_{I}I_{t}+\gamma_{H}H_{t}-\mu A_{t}\\ E_{t}+\beta_{A}A_{t}I_{t}-\alpha E_{t}-\mu E_{t}\\ I_{t}+\alpha E_{t}-\gamma_{I}I_{t}-\eta I_{t}-\mu I_{t}\\ \begin{array}{l} H_{t}+\eta I_{t}-\gamma_{H}H_{t}-\kappa_{H}H_{t}-\mu H_{t}\\ D_{t}+\kappa_{H}H_{t} \end{array} \end{array}\right.$$

Linearising (28) through the first-order Taylor series expansion yields the Jacobian matrix ***F****_t_*:

(29) $${\left.\phi (x_{t})=\frac{\partial \phi \left(x_{t}\right)}{\partial x}\right|}_{x_{t}=x_{t|t-1}}\approx F_{t}x_{t}$$

where

(30) $$F_{t}=\left[\begin{array}{ccccc} 1-\beta_{A}I_{t}-\mu & 0 & -\beta_{A}A_{t}+\gamma_{1} & \gamma_{H} & 0\\ \beta_{A}I_{t} & 1-\left(\alpha +\mu \right) & \beta_{A}A_{t} & 0 & 0\\ 0 & \alpha & 1-\left(\gamma_{I}+\eta +\mu \right) & 0 & 0\\ 0 & 0 & \eta & 1-\left(\gamma_{H}+\kappa_{H}+\mu \right) & 0\\ 0 & 0 & 0 & \kappa_{H} & 1 \end{array}\right]$$

Thus, (30) can be rewritten as

(31) $$x_{t}=F_{t}x_{t-1}$$

As observed data for active cases, hospital admissions and deceased cases can be obtained from the current public report [1]. The observation vector can be defined as ***z****_t_* = [*I_t_*, *H_t_*, *D_t_*]^T^, and the observation equation yields

(32) $$z_{t}=\bar{\Gamma }x_{t}$$

where

(33) $$\bar{\Gamma }=\left[\begin{array}{ccccc} 0 & 0 & 1 & 0 & 0\\ 0 & 0 & 0 & 1 & 0\\ 0 & 0 & 0 & 0 & 1 \end{array}\right]$$

In order to identify the time-varying parameters, we augment the model parameter vector ***θ*** = [*β_A_*, *γ_I_*, *γ_H_*]^T^ into ***x****_t_*, which yields ***X****_t_* = [***x****_t_*, ***θ****_t_*] and ***Φ****_t_* = diag(***F****_t_*, ***I***), with a 3 × 3 identity matrix ***I***.

Consequently, we have the system for parameter and state estimation, shown as

(34) $$\left\{\begin{array}{c} X_{t+1}=\Phi_{t}X_{t}+w_{k}\\ z_{t}=\Gamma X_{t}+v_{t} \end{array}\right.$$

where ***w****_t_* is the system process noise, ***v****_k_* is the observation noise, and

(35) $$\Gamma =\left[\begin{array}{ll} \bar{\Gamma } & 0_{3\times 3} \end{array}\right]$$

By mapping the transition matrix path noise through the Jacobian, the noise propagates into the state, forming an 8 × 8 non-diagonal covariance matrix:

(36) $$Q=\left[\begin{array}{cccccc} q_{1}+q_{4}+q_{5} & -q_{1} & -q_{4} & -q_{5} & 0 & 0\\ -q_{1} & q_{1}+q_{2} & -q_{2} & 0 & 0 & 0\\ -q_{4} & -q_{2} & q_{2}+q_{3}+q_{4} & -q_{3} & 0 & 0\\ -q_{5} & 0 & -q_{3} & q_{3}+q_{5}+q_{6} & -q_{6} & 0\\ 0 & 0 & 0 & -q_{6} & q_{6} & 0\\ 0 & 0 & 0 & 0 & 0 & \ddots \end{array}\right]$$

where $q_{1}$, $q_{2}$, $q_{3}$, $q_{4}$, $q_{5}$, and $q_{6}$ denote the noises of transitions from *A*→*E*, *E*→*I*, *I*→*H*, *I*→*A*, *H*→*A*, and *H*→*D*. These noises of transitions are acquired using the state-dependent noise approach [18], where each transition noise reflects the variability in the corresponding compartment transitions. The observation noises are subject to a zero-mean Gaussian distribution with corresponded covariances, i.e., $E[v_{t}{v_{t}}^{T}]=R$.

We can estimate the state and parameters by the following procedure:

***Step 1***: Initialise the predicted state ${\hat{X}}_{0}$ and its corresponding covariance ${\hat{P}}_{0}$:

(37) $$\left\{\begin{array}{l} {\hat{X}}_{0}=E[X_{0}]\\ {\hat{P}}_{0}=E[(X_{0}-{\hat{X}}_{0}){\left(X_{0}-{\hat{X}}_{0}\right)}^{T}] \end{array}\right.$$

***Step 2***: Predict the prior state estimation ${\hat{X}}_{t}^{-}$ and its corresponding covariance ${\hat{P}}_{t}^{-}$:

(38) $${\hat{X}}_{t}^{-}=\Phi_{t}X_{t-1}$$

(39) $${\hat{P}}_{t}^{-}=\Phi_{t}^{-}{\hat{P}}_{t-1}{\Phi_{t}^{-}}^{T}+Q_{t}$$

***Step 3***: Calculate the Kalman gain based on the joint error covariance of the state and observation:

(40) $$K_{t}={\hat{P}}_{t}^{-}\Gamma^{T}{\left(\Gamma {\hat{P}}_{t}^{-}\Gamma^{T}+R\right)}^{-1}$$

***Step 4***: Correct the predicted state and its error covariance:

(41) $${\hat{X}}_{t}={\hat{X}}_{t}^{-}+K_{t}\left({\hat{z}}_{t}-\Gamma {\hat{X}}_{t}^{-}\right)$$

(42) $${\hat{P}}_{t}=\left(I-K_{t}\Gamma \right){\hat{P}}_{t}^{-}{\left(I-K_{t}\Gamma \right)}^{T}$$

***Step 5***: Repeat (38)~(42) for all samples.

## 3. Performance Evaluation and Discussion

Simulations were conducted to assess the performance of the EKF estimation based on the proposed AEIHD model (EKF-AEIHD). The assessment focused on the following perspectives: (i) the effectiveness of the proposed AEIHD model compared to the classical SEIRD model; (ii) the estimation accuracy of EKF versus Markov chain Monte Carlo (MCMC) when applied to the AEIHD model; and (iii) a comparison of the estimates obtained from both the SEIRD and AEIHD models, using the MCMC and EKF methods, against actual reported data.

To evaluate the accuracy of the estimation, the root mean square error (RMSE) is employed, which is defined as

(43) $$\mathrm{RMSE}=\sqrt{\frac{1}{n}\overset{n}{\underset{i=1}{\sum }}{\left({\hat{x}}_{i}-x_{i}^{ref}\right)}^{2}}$$

where *x_i_^ref^* denotes the reference data for size *n*.

### 3.1. Numerical Solution of AEIHD Model

Simulations were conducted to evaluate the proposed AEIHD model’s performance in analysing the dynamics of transmission in varying post-COVID-19 circumstances. For the simulation setup, the initial population size was set to one million individuals. According to global statistics [1], the number of natural births was set to 40 per simulation time step, and the natural death rate was set to 5‱ for the entire simulation period. The remaining simulation parameters for the AEIHD model are shown in Table 1. The numerical solutions of SEIRD were also obtained to evaluate the effectiveness of the proposed AEIHD model in the post-COVID-19 era. The SEIRD parameters and initial transmission states were referenced from [18]. The simulation was conducted within a 300-day period to demonstrate the transmission pattern in the context of a long-term pandemic, considering the effects of antibodies’ effectiveness and virus mutations.

**Figure 3:**
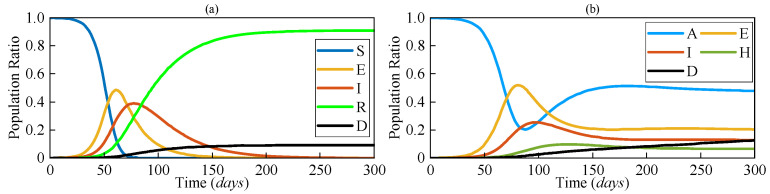
The population ratios of each compartment based on (**a**) SEIRD and (**b**) AEIHD.

The numerical solutions of both SEIRD and AEIHD were calculated by the fourth-order Runge–Kutta method and are shown in Figure 3, demonstrating the population ratio transmission of each compartment for both models under a similar scenario. Although the rapid descent in the susceptible compartment in the early stage explains the viral spread of COVID-19, the number of susceptible individuals being reduced to zero after 70 days is not realistic because the antibodies’ effectiveness is not lifelong. In contrast, the joint antibody-acquired compartment suggested by the proposed AEIHD model makes a realistic hypothesis that individuals who were vaccinated or recovered from the disease still potentially become infected due to vaccination ineffectiveness or virus variants.

As shown in Figure 3, the number of people in the *A* compartment will not decrease to zero after the outbreak, as they will gain immunity from recovery. Correspondingly, this limited duration of antibody effectiveness makes the risk of an outbreak remain for an extremely long time, resulting in continuous growth in fatalities at a relatively small rate. In the post-COVID-19 era, the public healthcare system has been able to guarantee sufficient treatment for patients with severe symptoms and collect accurate reports from hospitals, compensating for the absence of large-scale screening. Therefore, modelling the hospitalised compartment is consistent with community interests and helps allocate healthcare resources more efficiently to cope with the onslaught of recurring outbreaks at any time. In order to analyse the influence of *A* and *H*, the numerical solutions with different *β_A_* and *η* were further evaluated, respectively.

**Figure 4:**
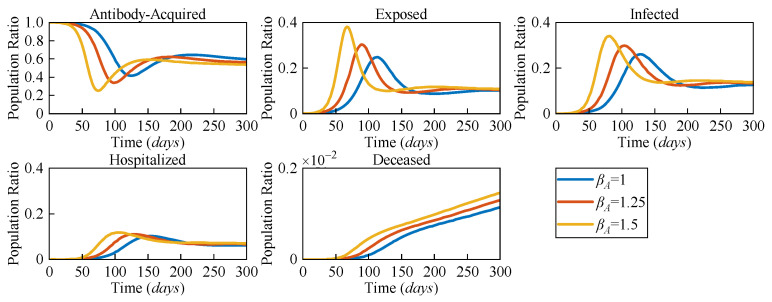
The population ratios of all compartments of the AEIHD model at three different antibody-acquired infection rates (*β_A_* = 0.75, *β_A_* = 1, and *β_A_* = 1.5).

Figure 4 illustrates the numerical solutions of each compartment of the AEIHD model at various antibody-acquired infection rates (*β_A_* = 0.75, *β_A_* = 1, *β_A_* = 1.5). The intensity of virus transmission will vary with virus mutation, leading to a change in the infection rate, which further impacts the dynamics of COVID-19 transmission. Therefore, we set three distinct infection rates, *β_A_*, to evaluate their impact on transmission in terms of all compartments in the community. The results indicate that the larger *β_A_*, the earlier the antibody-acquired population begins to decline, which occurs on Days 43, 31, and 25, respectively. These descents reach their lowest ratios of 0.42, 0.34, and 0.25 by Days 128, 100, and 73, correspondingly. This indicates that a higher infection rate induces a larger scale of exposure to potential risks when the immunisation barrier is challenged. Correspondingly, the number of exposed and infected individuals will increase to 0.39 and 0.376 at *β_A_* = 1.5, respectively, which are greater than the other given *β_A_* values. Furthermore, hospitalisations and deceased individuals reach a higher peak. Therefore, we can infer that as the virus mutates intermittently, the risk of an outbreak remains in the community even when antibody-acquired persons are the majority.

**Figure 5:**
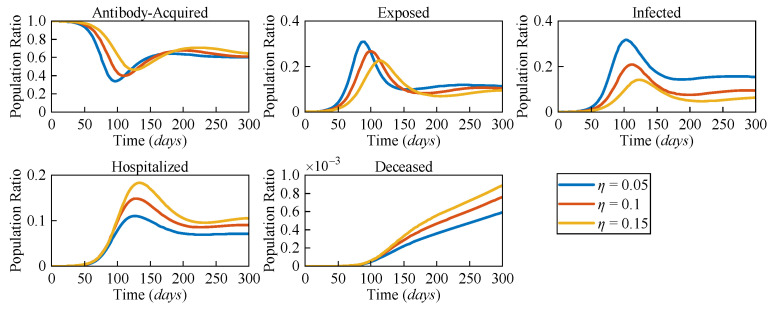
The population ratios of all compartments of the AEIHD model at three different severe symptomatic rates (*η* = 0.05, *η* = 0.1, and *η* = 0.15).

Due to the distinct pathological characteristics among different variants, the likelihood of severe symptoms changes accordingly. To assess the impact of varying risk levels on COVID-19 dynamics, three different severe symptomatic rates (*η* = 0.05, *η* = 0.1, and *η* = 0.15) were considered. Figure 5 presents the numerical solutions for all compartments in the AEIHD model at different *η*.

In the post-COVID-19 era, with the gradual relaxation of healthcare resource constraints, it is assumed that all severe cases can receive hospitalisation treatment. The results clearly show that as *η* increases, both the hospitalised and deceased populations rise significantly. Counterintuitively, an increase in the severe symptomatic rate delays the peak of the infected population while also reducing the corresponding peak value. Compared to the scenario with *η* = 0.05, the peak of the exposed population with *η* = 0.15 is delayed by 29 days, and the peak of the infected population is delayed by 24 days, with the population ratios decreasing by 0.056 and 0.133, respectively. This is because the larger η results in a higher proportion of infected individuals being isolated, thereby reducing their interactions with the general population. As a result, the overall transmission rate declines, causing a delayed and lower peak in both the exposed and infected populations. Through this analysis, the proposed AEIHD model highlights the capability of capturing the subtle relationship between viral variations and severe symptomatic rates.

By integrating antibody-acquired and hospitalised compartments, the model reflects the dynamic interplay of reinfections and severe cases, offering a nuanced understanding of how viral characteristics influence transmission dynamics. This makes the AEIHD model particularly well suited for guiding public health strategies in the post-COVID-19 era.

### 3.2. EKF Based on the AEIHD Model

To evaluate the EKF estimation performance based on the proposed AEIHD model, observational data were produced by introducing random Gaussian noise with the covariance ***Q*** = 0.01 into the AEIHD model’s numerical solution, as shown in Figure 3b, to mimic the actual reported data on COVID-19. The initial values for the transmission state and parameters are the same as those of the previous simulation.

**Figure 6:**
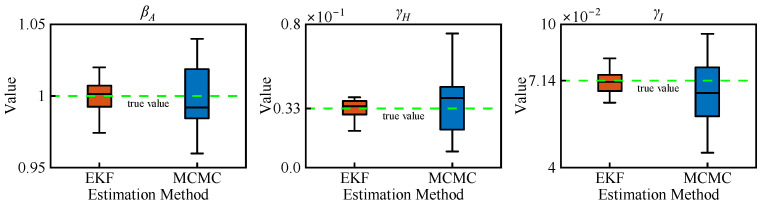
The estimation of parameters by MCMC (3000 samples) and EKF (300 days) based upon the AEIHD model.

Figure 6 illustrates the parameter estimation results by MCMC with 3000 samples and EKF over 300 days, with reference to their true values given in Table 1. It is obvious that the EKF estimation of the model parameters closely aligns with the reference while demonstrating higher precision compared to MCMC estimates, which exhibit greater variability around the true values. The RMSEs of the EKF estimates are about 0.0034, 0.0027, and 0.0025 for parameters *β_A_*, *γ_I_*, and *γ_H_*, respectively, as shown in Table 2. However, the RMSEs obtained using MCMC are about twice as large as those of EKF. Therefore, it is evident that the suggested EKF based on the AEIHD model can effectively estimate the model parameters.

**Figure 7:**
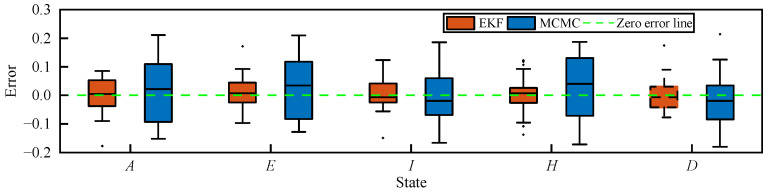
Estimation errors of the transmission state obtained by MCMC and EKF based on the AEIHD model.

Figure 7 presents the estimation errors of the transmission states of MCMC and EKF methods based on the AEIHD model. It is clear that the EKF estimations of all compartments closely approximate their true values. The RMSEs of all compartments obtained by EKF are 4.1%, 3.2%, 3.8%, 2.9%, and 3.9%, as indicated in Table 3, which are approximately two to three times lower than those of MCMC. Therefore, the suggested EKF based upon the AEIHD model can effectively estimate the transmission state and model parameters.

### 3.3. COVID-19 Spread in Australia

Simulation trials were also conducted to examine the effectiveness of the proposed EKF-AEIHD for the COVID-19 spread in Australia. As an island nation with well-documented pandemic data, Australia serves as a valuable case study for assessing epidemic models. Comprehensive COVID-19 data from Australia were used as a reference for simulation analysis, allowing for a detailed examination of the proposed method’s performance in real-world scenarios. The 300-day simulation period, spanning from 29 December 2021 (Day 1), to 25 October 2022 (Day 300), began with over 23 million Australians vaccinated, representing more than 85% of the total population. During this period, four COVID-19 outbreaks were recorded, lasting 64, 65, 41, and 130 days, respectively. These outbreaks were likely driven by new Omicron subvariants (e.g., BA.2, BA.4, and BA.5) and the waning immunity among individuals with existing antibodies, leading to reinfections among individuals with existing antibodies.

For the simulation analysis, observational data were collected from the online database [1] of daily reported COVID-19 statistics in Australia throughout the entire simulation period. Given the absence of true values, the reported data served as the reference for calculating the estimation error. The initial transmission states and model parameters of the AEIHD model are given in Table 4 and Table 5. The covariance matrix ***Q*** was obtained using the state-dependent covariance approach [18], and ***R*** was obtained from the variances of observation errors in the reported data for infected, hospitalised, and deceased cases. To facilitate comparison, simulation trials were performed using three schemes: MCMC based on the SEIRD and AEIHD models (referred to as MCMC-SEIRD and MCMC-AEIHD) and the proposed EKF-AEIHD (the state estimate after the Kalman correction). A 100-day (Days 201–300) forecast of infection cases using the EKF-AEIHD method was conducted, excluding infection case updates and relying only on hospitalisation and death data for observations. Similar to [18], the MCMC sample size was set to 30 per day for both AEIHD and SEIRD to enable online estimation performance. For the initial transmission states of the SEIRD model, *E*(0), *I*(0), and *D*(0) are set to match those of the AEIHD model, while *R*(0) is approximated from cumulative infections. *S*(0) is then determined by subtracting *E*(0), *I*(0), *R*(0), and *D*(0) from the total population.

**Figure 8:**
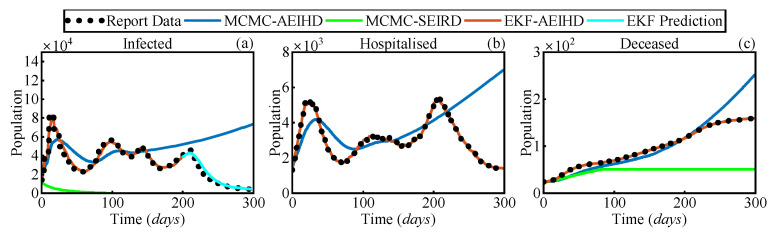
Estimated transmission states by MCMC-SEIRD, MCMC-AEIHD, and proposed EKF-AEIHD: (**a**) infected population: 200-day estimation and 100-day prediction, (**b**) hospitalised population, and (**c**) deceased population.

Figure 8 shows the estimated infected, hospitalised, and deceased populations using MCMC-SEIRD and MCMC-AEIHD over 300 days, while EKF-AEIHD estimates hospitalised and deceased cases for 300 days and forecasts infected cases for the last 100 days following a 200-day estimation period. Notably, MCMC-SEIRD only provides estimates for the infected and deceased populations, as the SEIRD model does not include a hospitalised compartment. As shown in Figure 8a,c, MCMC-SEIRD estimates that the infected population rapidly decreases from Day 1 and drops to zero before Day 100, resulting in the deceased population remaining stable at 49 over the subsequent period. This outcome is due to the traditional model’s assumption of lifelong immunity following recovery and a $R_{0}$ value below the threshold, which ultimately leads the system to converge to the disease-free equilibrium. In contrast, the methods based on the AEIHD model produce dynamics that closely align with the reported infected population. Further, the disease-free equilibrium of the system was not achieved, as $R_{0}>1$, evaluated from (15), remained above the threshold, indicating sustained transmission within the population. This suggests that the AEIHD model provides a more accurate representation of COVID-19 dynamics in the post-pandemic era. However, due to the cumulative error in MCMC-AEIHD, its estimates significantly deviate after Day 139 because the MCMC algorithm assumes constant parameters, limiting its adaptability to dynamic changes. The EKF-AEIHD improves accuracy by incorporating state and covariance corrections, allowing the parameters corresponding to the AEIHD model to adapt dynamically based on observations from Day 0 to Day 200. Building on this, after Day 200, the infection cases are not included in the observations, and only hospitalised and deceased data are used to propagate the system forward, yielding a 100-day prediction of infected cases. The results present that the predicted results align well with reported data, demonstrating that our method successfully calibrates infection estimates and maintains at least 100 days of estimation stability despite missing infection observations. To further assess the accuracy of the proposed EKF-AEIHD method, the RMSEs for the infected, hospitalised, and deceased populations were statistically evaluated against the reported data. As shown in Table 6, the RMSEs of MCMC-AEIHD are 27.4% for infections, 32.8% for hospitalisations, and 28.9% for deceased, whereas EKF-AEIHD achieves significantly lower RMSEs of 6.9% (estimation) and 9.5% (prediction) for infections, 5.8% for hospitalisations, and 6.1% for deceased. Due to the significant divergence of MCMC estimates, the RMSE of MCMC-SEIRD is approximately 12 times larger than that of EKF-AEIHD. The results indicate that EKF-AEIHD effectively captures the dynamics of epidemic transmission using limited reported data. Additionally, the proposed method accurately forecasts the 100-day infection cases. This makes it especially suitable for forecasting COVID-19 spread in the post-pandemic era and modelling other infectious diseases with non-lifelong immunity characteristics.

This makes it especially suitable for forecasting COVID-19 spread in the post-pandemic era and modelling other infectious diseases with non-lifelong immunity characteristics.

**Figure 9:**
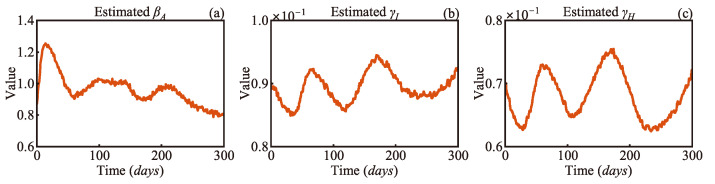
Estimated parameters by EKF: (**a**) antibody-acquired infection rate *β_A_*, (**b**) infection recovery rate *γ_I_*, and (**c**) hospitalised recovery rate *γ_H_*.

Figure 9 presents the EKF-estimated variations in the antibody-acquired infection rate, infection recovery rate, and hospitalised recovery rate over 300 days. As shown in Figure 9a the trend in *β_A_* aligns well with the infection trend illustrated in Figure 8a indicating that the transmission rate during this period closely corresponds to the actual infection dynamics. During this time, the main strain circulating in the community was Omicron and its variants. Even though most people had developed antibodies, infections continued to occur, reflecting the fact that Omicron’s immune escape capability weakened the protective effect of immunity. As shown in Figure 9b *γ_I_* displays a trend that is almost the opposite of *β_A_*. A reasonable inference is that, as the infection risk rises and the number of infected individuals grows, the increased short-term pressure on healthcare resources leads to a decrease in recovery rates. This phenomenon underscores the importance of the real-time monitoring of recovery rates for optimising the allocation of medical resources. Figure 9c displays a trend similar to that of *γ_I_*. However, during the third outbreak, *γ_H_* dropped to a lower level than it did during the previous two outbreaks. The likely reason is that the Australian government’s relaxation of COVID-19 restrictions during this period increased population mobility, further exacerbating the potential for virus transmission. Additionally, the end of large-scale screening led to significant underreporting of infections. Meanwhile, hospitalisation data remained a more reliable indicator of the true spread of the virus. These findings highlight the importance of hospitalisation-based tracking in the post-COVID-19 era, as it provides a more realistic measure of disease transmission when widespread testing is no longer available.

## 4. Conclusions

This paper addresses the challenges posed by variant viruses in the post-COVID-19 era for modelling epidemic dynamics. Its main contributions are as follows: (I) the proposed AEIHD model removes the susceptible compartment and integrates the recovered and vaccinated populations into a unified “antibody-acquired” compartment; (II) a hospitalised compartment is introduced to handle incomplete data in the post-COVID-19 era and inform medical resource allocation; and (III) EKF-AEIHD is employed to enhance the precision of real-time state and parameter estimations. The proposed AEIHD model and its integration with EKF estimation provide a reliable and accurate approach to epidemic modelling, offering valuable insights for public health strategies and resource planning, particularly in the post-COVID-19 era.

Future improvements in the proposed method include incorporating stochastic differential equations to address data randomness and delays. Further, incorporating detailed population stratification, such as age groups, environmental factors, and vaccine types, would improve the model’s capacity to represent heterogeneous immunity and the dynamics of disease transmission. By addressing these limitations, it is expected that the proposed approach will further enhance the reliability of COVID-19 prediction for public health decision-making and resource allocation when facing infectious disease threats.

## Figures and Tables

**Table 1:** Simulation parameter settings.

Parameter	Describe	Value
Λ	Natural birth rate	40 persons/day
*μ*	Natural death rate	5‱ for entire simulation period
*β_A_*	Antibody-acquired transmission rate	1/*N*_0_
*α*	Rate of exposed individuals who develop symptoms	0.143
*η*	Severe symptomatic rate	0.125
*γ_I_*	Infected patient recovery rate	0.0714
*γ_H_*	Hospitalised patient recovery rate	0.033
*κ_H_*	Hospitalised decease rate	0.05
Simulation time		300 days
Time step		1 day

**Table 2:** RMSEs of the estimated parameters using MCMC and EKF.

	*β_A_*	*γ_I_*	*γ_H_*
EKF	3.4 × 10^−3^	2.7 × 10^−3^	2.5 × 10^−3^
MCMC	7.1 × 10^−3^	7.2 × 10^−3^	6.6 × 10^−3^

**Table 3:** RMSEs of the estimated states using MCMC and EKF.

	*A*	*E*	*I*	*H*	*D*
EKF	4.1%	3.2%	3.8%	2.9%	3.9%
MCMC	14.2%	13.7%	10.8%	13.4%	8.7%

**Table 4:** The initial values of the transmission states for the COVID-19 pandemic in Australia.

State	Value (*Individuals*)	Source
*A*(0)	23 million	MCMC
*E*(0)	49,673	MCMC
*I*(0)	14,252	[1]
*H*(0)	1310	[1]
*D*(0)	24	[1]

**Table 5:** The initial parameters for the COVID-19 pandemic in Australia.

Parameter	Value	Source
Λ(0)	839 ppl/day	[29]
*μ*(0)	6.7‱	[30]
*β_A_*(0)	0.89	MCMC
*α*(0)	0.11	[22]
*η*(0)	0.16	MCMC
*γ_I_*(0)	0.11	MCMC
*γ_H_*(0)	0.06	MCMC
*κ_H_*(0)	0.0027	MCMC

**Table 6:** RMSEs of MCMC-SEIRD, MCMC-AEIHD, and EKF-AEIHD for the Australia COVID-19 spread.

Compartment	EKF Based on AEIHD Model	MCMC Based on AEIHD Model	MCMC Based on SEIRD Model
Infected	6.9% (estimation) and 9.5% (prediction)	27.4%	94.5%
Hospitalised	5.8%	32.8%	-
Deceased	6.1%	28.9%	61.2%

## Data Availability

The data are available from the lead author upon reasonable request.

## References

[1] Mathieu E., Ritchie H., Rodés-Guirao L., Appel C., Giattino C., Hasell J., Bobbie M., Dattani S., Beltekian D., Ortiz-Ospina E. Coronavirus Pandemic (COVID-19). https://github.com/owid/covid-19-data/tree/master/public/data.

[2] Coccia M. (2023). Sources, Diffusion and Prediction in COVID-19 Pandemic: Lessons Learned to Face Next Health Emergency. AIMS Public Health.

[3] Caldwell J.M., Le X., McIntosh L., Meehan M.T., Ogunlade S., Ragonnet R., O’Neill G.K., Trauer J.M., McBryde E.S. (2021). Vaccines and Variants: Modelling Insights into Emerging Issues in COVID-19 Epidemiology. Paediatr. Respir. Rev..

[4] Jacobs J.L., Haidar G., Mellors J.W. (2023). COVID-19: Challenges of Viral Variants. Annu. Rev. Med..

[5] Al-Hatamleh M.A., Abusalah M.A., Ma’mon M.H., Alshaer W., Ahmad S., Mohd-Zahid M.H., Rahman E.N.S.E., Yean C.Y., Alias I.Z., Uskoković V. (2023). Understanding the Challenges to COVID-19 Vaccines and Treatment Options, Herd Immunity and Probability of Reinfection. J. Taibah Univ. Med. Sci..

[6] Cuevas E. (2020). An Agent-Based Model to Evaluate the COVID-19 Transmission Risks in Facilities. Comput. Biol. Med..

[7] Chang S.L., Harding N., Zachreson C., Cliff O.M., Prokopenko M. (2020). Modelling Transmission and Control of the COVID-19 Pandemic in Australia. Nat. Commun..

[8] Kermack W.O., McKendrick A.G. (1991). Contributions to the Mathematical Theory of Epidemics-I. 1927. Bull. Math. Biol..

[9] Kuhl E. (2021). Data-driven network SEIR model. Computational Epidemiology: Data-Driven Modelling of COVID-19.

[10] Frank T.D. (2022). COVID-19 Epidemiology and Virus Dynamics: Nonlinear Physics and Mathematical Modeling.

[11] Dashtbali M., Mirzaie M. (2021). A Compartmental Model That Predicts the Effect of Social Distancing and Vaccination on Controlling COVID-19. Sci. Rep..

[12] Badr H.S., Du H., Marshall M., Dong E., Squire M.M., Gardner L.M. (2020). Association between Mobility Patterns and COVID-19 Transmission in the USA: A Mathematical Modelling Study. Lancet Infect. Dis..

[13] Ukwishaka J., Ndayishimiye Y., Destine E., Danwang C., Kirakoya-Samadoulougou F. (2023). Global Prevalence of Coronavirus Disease 2019 Reinfection: A Systematic Review and Meta-Analysis. BMC Public Health.

[14] Li Z., Yi Y., Luo X., Xiong N., Liu Y., Li S., Sun R., Wang Y., Hu B., Chen W. (2020). Development and Clinical Application of a Rapid Igm-Igg Combined Antibody Test for Sars-Cov-2 Infection Diagnosis. J. Med. Virol..

[15] Kamińska D., Dęborska-Materkowska D., Kościelska-Kasprzak K., Mazanowska O., Remiorz A., Poznański P., Durlik M., Krajewska M. (2022). Immunity after COVID-19 Recovery and Vaccination: Similarities and Differences. Vaccines.

[16] Gousseff M., Penot P., Gallay L., Batisse D., Benech N., Bouiller K., Collarino R., Conrad A., Slama D., Joseph C. (2020). Clinical Recurrences of COVID-19 Symptoms after Recovery: Viral Relapse, Reinfection or Inflammatory Rebound. J. Infect..

[17] Mummert A., Otunuga O.M. (2019). Parameter Identification for a Stochastic Seirs Epidemic Model: Case Study Influenza. J. Math. Biol..

[18] Zhu X., Gao B., Zhong Y., Gu C., Choi K.-S. (2021). Extended Kalman Filter Based on Stochastic Epidemiological Model for COVID-19 Modelling. Comput. Biol. Med..

[19] Zhu X., Yuanyou S., Zhong Y. (2024). An EKF Prediction of COVID-19 Propagation under Vaccinations and Viral Variants. Math. Comput. Simul..

[20] Piccirillo V. (2021). Nonlinear Control of Infection Spread Based on a Deterministic Seir Model. Chaos Solitons Fractals.

[21] Song J., Xie H., Gao B., Zhong Y., Gu C., Choi K.-S. (2021). Maximum Likelihood-Based Extended Kalman Filter for COVID-19 Prediction. Chaos Solitons Fractals.

[22] Sameni R. (2020). Mathematical Modeling of Epidemic Diseases; a Case Study of the COVID-19 Coronavirus. arXiv.

[23] Singh K.K., Kumar S., Dixit P., Bajpai M.K. (2021). Kalman Filter Based Short Term Prediction Model for COVID-19 Spread. Appl. Intell..

[24] Silveira A., Pereira A. (2020). Estimation and monitoring of COVID-19′s transmissibility from publicly available data. Front. Appl. Math. Stat..

[25] Arroyo-Marioli F., Bullano F., Kucinskas S., Rondón-Moreno C. (2021). Tracking R of COVID-19: A New Real Time Estimation Using the Kalman Filter. PLoS ONE.

[26] Alyami L., Panda D.K., Das S. (2023). Bayesian Noise Modelling for State Estimation of the Spread of COVID 19 in Saudi Arabia with Extended Kalman Filters. Sensors.

[27] Chatterjee K., Chatterjee K., Kumar A., Shankar S. (2020). Healthcare Impact of COVID-19 Epidemic in India: A Stochastic Mathematical Model. Med. J. Armed Forces India.

[28] He S., Tang S., Rong L. (2020). A Discrete Stochastic Model of the COVID-19 Outbreak: Forecast and Control. Math. Biosci. Eng..

[29] Australian Bureau of Statistics (2023). Births, Australia. https://www.abs.gov.au/statistics/people/population/births-australia/latest-release.

[30] Australian Bureau of Statistics (2023). Deaths, Australia. https://www.abs.gov.au/statistics/people/population/deaths-australia/latest-release#cite-window1.

